# Fever Inspiration: Precision Engineering for Safe and Systemic Immunotherapy

**DOI:** 10.1002/advs.202513566

**Published:** 2025-11-26

**Authors:** Yang Li, Fulong Man, Jianwei Cheng, Li Chen, Yongyong Li, Haiqing Dong

**Affiliations:** ^1^ Key Laboratory of Spine and Spinal Cord Injury Repair and Regeneration Ministry of Education Tongji Hospital Department of Pharmacy School of Medicine Tongji University Shanghai 200065 China; ^2^ Shanghai Skin Disease Hospital The Institute for Biomedical Engineering and Nano Science (iNANO) School of Medicine Tongji University Shanghai 200331 China

**Keywords:** engineered fever therapy, fevers, nanomaterials, thermal immunotherapy

## Abstract

Fever, a conserved physiological response, orchestrates robust immunomodulation with significant therapeutic potential. Engineered fever therapy (EFT) leverages this mechanism to activate systemic immune response, emerging as a transformative adjuvant strategy for disease management, particularly in oncology. However, achieving precise and safe EFT remains challenging, largely due to unpredictable temperature fluctuations and inconsistent immune responses. To address these limitations, thermal immunotherapy integrates precision thermal modulation with nanotechnology, enabling targeted induction of fever‐range temperatures (38.5–40 °C) within tumor sites. Building upon physiological fever mechanisms and precision bioengineering, controllable EFT establishes a new paradigm with three core principles: i) precise fever induction with input and process control, ii) quantitative reprogramming of immune pathways, and iii) enhanced safety through optimized fever dynamics. This review comprehensively summarizes the molecular and physiological underpinnings of fever, evaluates its therapeutic potential in disease management, and presents recent progress in thermal immunotherapy and the EFT. Furthermore, future perspectives for controllable EFT are discussed and critically assessed, along with its opportunities and main challenges for clinical translation in precision oncology.

## Graphical abstract

This review examines engineered fever therapy (EFT) as a novel cancer treatment, harnessing fever's immunomodulatory effects to enhance systemic immune responses. It explores molecular mechanisms, advances in nanotechnology‐driven thermal immunotherapy, and a bioengineered framework for precise, safe fever induction. The paper evaluates EFT's potential and challenges for clinical translation in precision oncology.

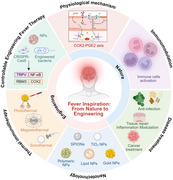

## 1 Introduction

Cancer represents a formidable global public health challenge, characterized by its high incidence and mortality rates.^[^ ^1^ ^]^ According to the International Agency for Research on Cancer, ≈20 million new cancer cases and 9.7 million cancer‐related deaths were reported worldwide in 2022.^[^ ^2^ ^]^ Despite advances in conventional surgery, chemotherapy, and radiotherapy, their clinical efficacy is still limited by systemic toxicity, residual tumors, and uncontrolled metastases.^[^ ^3^ ^]^ Metastatic tumors, the leading cause of cancer mortality, remain particularly refractory to current interventions, underscoring a critical unmet need in oncology.^[^ ^4^, ^5^ ^]^ Thus, the development of precision therapies capable of addressing both primary and metastatic disease has become an urgent imperative.

Fever is a systemic immune response to infection that plays a crucial role in immunomodulation as an evolutionarily conserved physiological mechanism.^[^ ^6^, ^7^ ^]^ Briefly, fever activates innate and adaptive immune responses through multiple mechanisms, including enhanced migration and activation of immune cells (e.g., macrophages and natural killer (NK) cells) and the release of pro‐inflammatory cytokines.^[^ ^8^, ^9^, ^10^, ^11^, ^12^ ^]^ Given the immunomodulatory effects of fever, its therapeutic significance is increasingly recognized, particularly in cancer therapy.^[^ ^13^, ^14^, ^15^ ^]^ EFT represents a bioengineered approach to induce systemic fever, elevating core body temperature to enhance immune‐mediated disease management. Drawing on physiological mechanisms of fever, EFT uses pathogen‐associated molecular pattern (PAMP)‐based drugs or bacterial agents to elevate core body temperature and thereby activate a systemic immune response. This approach has demonstrated efficacy in combating infections and tumors.^[^ ^9^, ^13^, ^16^, ^17^, ^18^, ^19^, ^20^, ^21^ ^]^ For example, EFT utilizing PAMP‐based agents, such as Iscador or Picibanil, triggers a broad immune response, potentiating dendritic cell (DC) maturation and driving T‐helper 1 (Th1) and cytotoxic T lymphocyte (CTL) activation.^[^ ^15^, ^22^ ^]^ These immunomodulatory effects suppress tumor cell proliferation and migration, offering therapeutic efficacy against infectious diseases and cancer. However, current fever induction methods are constrained by the risks of uncontrolled hyperthermia and associated safety concerns, blocking the clinical applicability of EFT.^[^ ^15^, ^23^, ^24^ ^]^

Recent advancements in thermal immunotherapy encompass modalities such as local photothermal therapy, magnetic hyperthermia, and hot bath therapy.^[^ ^25^, ^26^ ^]^ In contrast to EFT's whole‐body fever, thermal immunotherapy delivers localized hyperthermia (>42 °C) to pathological tissues to enhance immune cell activity and eliminate aberrant cells.^[^ ^26^ ^]^ Nanomedicine‐based thermal immunotherapy stands out for its precise spatiotemporal control, enabling tunable modulation of thermal distribution, duration, and intensity through optimized stimulation parameters.^[^ ^27^, ^28^, ^29^ ^]^ These advancements offer critical insights into overcoming EFT's limitations, paving the way for controllable EFT strategies that integrate input/process control, quantitative immune reprogramming, and optimized fever dynamics.

This review elucidates the molecular and physiological mechanisms underpinning the therapeutic potential of fever, summarizes recent progress in nanomedicine‐based thermal immunotherapy, and proposes a bioengineered framework for controllable EFT. The opportunities and challenges of EFT clinical translation are critically evaluated, highlighting its prospective role as a transformative adjuvant in precision cancer therapy.

## 2 Natural Fever

### 2.1 Physiological Mechanism of Natural Fever

**Figure 1:**
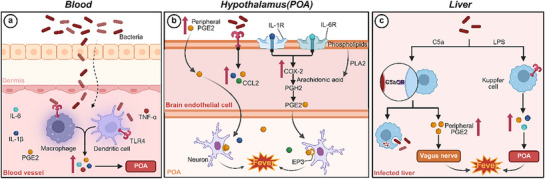
Fever mechanism during infection. Created in https://BioRender.com.

Fever arises from pyrogenic agents activating endogenous pyrogens (EPs)‐producing cells to generate and release EPs, orchestrated by complex interactions among the immune, neuronal, and endocrine systems.^[^ ^6^, ^21^, ^30^ ^]^ The process involves the activation of immune cells, release of EPs, and synthesis of prostaglandins (**Figure** **1**).^[^ ^31^, ^32^, ^33^, ^34^, ^35^, ^36^ ^]^

The hypothalamus serves as the central regulator of thermal homeostasis, orchestrating a dynamic balance between heat production and dissipation to maintain physiological body temperature.^[^ ^21^, ^37^, ^38^ ^]^ During infection or stress, peripheral immune cells, such as macrophages and DCs (Figure 1a), release prostaglandin E2 (PGE2) and EPs, including interleukin‐1β (IL‐1β), interleukin‐6 (IL‐6), and tumor necrosis factor‐ɑ (TNF‐ɑ), which activate hypothalamic thermoregulatory pathways to elevate body temperature.^[^ ^21^, ^31^, ^39^ ^]^ Meanwhile, blood–brain barrier endothelial cells secrete cytokines and chemokines (e.g., C─C motif ligand 2 (CCL2) and C─X─C motif chemokine ligand 8 (CXCL8)), stimulating neurons in the ventromedial preoptic area of the hypothalamus to raise the thermoregulatory set point (Figure 1b).^[^ ^35^ ^]^

PGE2 acts as the primary mediator of fever induction, coordinating a cascade of molecular events.^[^ ^33^, ^40^ ^]^ The fever process commences when toll‐like receptors (TLRs) or other pattern recognition receptors detect PAMPs (e.g., LPS), triggering the nuclear factor‐κB (NF‐κB)‐mediated synthesis of EPs.^[^ ^7^, ^31^, ^41^ ^]^ These cytokines, whether of systemic or local origin, subsequently upregulate cyclooxygenase‐2 (COX‐2) expression in brain endothelial cells.^[^ ^21^, ^31^, ^42^ ^]^ COX‐2 then catalyzes the conversion of arachidonic acid, liberated from membrane phospholipids by phospholipase A2 (PLA2), into prostaglandin H2 (PGH2).^[^ ^33^, ^43^, ^44^, ^45^ ^]^ PGH2 is then rapidly isomerized by terminal prostaglandin E synthases to produce PGE2 (Figure 1b).^[^ ^46^, ^47^, ^48^ ^]^ Within the hypothalamic preoptic area (POA), PGE2 binds to its EP3 receptor, elevating the thermoregulatory set point and initiating the release of norepinephrine and acetylcholine.^[^ ^49^, ^50^, ^51^ ^]^ Among them, norepinephrine promotes thermogenesis in brown adipose tissue and peripheral vasoconstriction to conserve heat, while acetylcholine induces skeletal muscle contractions, generating heat through shivering.^[^ ^7^, ^21^, ^52^, ^53^, ^54^ ^]^

In parallel, a complement‐mediated pathway, that is, the C5a‐CR pathway, provides a rapid COX‐2‐independent mechanism for fever induction, particularly during early infection (Figure 1c).^[^ ^55^, ^56^ ^]^ LPS activates the complement system, prompting hepatic Kupffer cells (KCs, specialized phagocytes in the liver) to produce peripheral PGE2, which either reaches the hypothalamus via systemic circulation or stimulates hepatic vagal afferents to signal thermoregulatory centers.^[^ ^30^, ^57^, ^58^ ^]^ KCs, as key immune effectors, filter circulating microbial products (e.g., Gram‐negative bacterial components) and serve as a primary source of EPs and PGE2, playing a pivotal role in fever onset.^[^ ^30^, ^32^, ^59^, ^60^ ^]^ The dual functionality underscores KCs’ potential as targets for precision interventions in controllable fever induction.

Despite these insights, critical gaps remain in fever regulation, particularly regarding the downstream pathways of PGE2‐EP3 receptor binding and the precise molecular cascades that connect fever to immune cell activation. A comprehensive elucidation of these molecular and cellular processes is essential to harness fever's therapeutic potential.

### 2.2 Immunomodulation by Fever

Elevated body temperature during fever enhances host defense by modulating immune cell activity, trafficking, and phenotypic polarization through intricate molecular signaling, cellular responses, tissue‐specific adaptations, and systemic immune coordination.^[^ ^19^, ^21^, ^61^, ^62^, ^63^, ^64^, ^65^, ^66^, ^67^, ^68^, ^69^, ^70^, ^71^, ^72^, ^73^, ^74^, ^75^, ^76^, ^77^, ^78^ ^]^ In this process, heat shock proteins (HSP70/90) and cytokines (IL‐1β/IL‐6/TNF‐α) serve as pivotal mediators, as summarized in **Table** **1**. Specifically, fever‐induced HSP70/90 drives macrophage adaptation,^[^ ^64^, ^68^ ^]^ T‐cell migration,^[^ ^74^ ^]^ and Th17 differentiation,^[^ ^62^ ^]^ whereas IL‐1β/IL‐6/TNF‐α enhances lymphocyte adhesion,^[^ ^19^ ^]^ Th1‐polarized^[^ ^72^ ^]^ and leukocyte exhibits promising clinical potential in oncology and infection control. FR‐WBH synergizes with fever‐induced responses to amplify lymphocyte and neutrophil responses while mitigating the adverse effects of bacterial infections.^[^ ^12^, ^19^, ^67^, ^68^, ^74^, ^78^, ^79^, ^81^ ^]^ These immunomodulatory effects demonstrate the therapeutic potential of controllable EFT, bridging innate and adaptive immunity to enhance disease management.

**Table 1:** Progress in the study of the immunomodulation effect of engineered fever and artificially mimicking the effects of natural fever.

Induction method	Temperature and duration	Research models	Involved cells	Immunomodulator markers	Regulatory effects
In vitro fever temperature exposure	39 and 41 °C for 2 h	Murine resident peritoneal macrophages	Macrophages	39–41 °C → FcR ↑, C3b ligands ↑ → Macrophage activation	39 °C: Macrophage functions enhanced (e.g., phagocytosis ↑) 41 °C: Functional suppression and induction of apoptosis.^[^ ^71^ ^]^
FR‐WBH	39.8 ± 0.2 °C for 6 h	BALB/c mice; human peripheral blood lymphocytes, mouse TK1 lymphoma cells	Lymphocytes, endothelial cells, tumor cells	39.8 °C → HEV ligand (PNAd/MAdCAM‐1) ↑ → Immune surveillance ↑; IL‐6, TNF‐α ↑; L‐selectin, α4β7 integrin ↑ → lymphocyte adhesion ↑	Immune surveillance ↑, lymphocyte adhesion ↑.^[^ ^19^ ^]^
FR‐WBH	39–40 °C for 6 h	BALB/c mice	Lymphocytes, alveolar macrophages	39–40 °C → HSP70, HSP110, GRP170 ↑ → Stress protein induction ↑	Cell survival ↑, Immune adaptation ↑.^[^ ^68^ ^]^
FR‐WBH	39.5–40 °C; Sustained for 24–72 h	CD‐1 mice, HSF‐1 null mice	Neutrophil, Macrophages	39.5–40 °C → IL‐1β, GM‐CSF ↑; CXCL2, CXCL1 ↑ → neutrophil recruitment ↑	Neutrophil recruitment ↑, respiratory burst ↑.^[^ ^78^ ^]^
In vitro fever temperature exposure	41 °C for 0.5 h; 41.5 °C for 1.5 h	Human monocyte‐derived DC	Monocyte, DCs	41–41.5 °C → IL‐12p70, TNF‐α ↑, IL‐10 ↓ (mature DCs) → Th1 polarization ↑ → IFN‐γ ↑, IL‐4 ↓; CCL19 ↑ → DC migration ↑	Th1 polarization ↑, DC migration ↑, and apoptosis ↓.^[^ ^72^ ^]^
In vitro fever temperature exposure	39.5 °C for 6 h	Human peripheral blood NK cells, Colo205 human colon adenocarcinoma cells, and colon tumor cells	NK cells, tumor cells	39.5 °C → NKG2D receptors ↑, MICA ligands ↑ → NK cell activation ↑	NK cytotoxicity ↑.^[^ ^10^ ^]^
FR‐WBH	39.5 °C for 6 h	Mice	Hematopoietic stem cells (HSCs), neutrophils, intestinal immune cells (e.g., Th17 cells, γδ T cells, paneth cells)	39.5 °C → IL‐1 ‐ IL‐17 ‐ G‐CSF axis ↑ → HSC proliferation and Neutrophil recovery ↑; CXCR4/CXCL12 ↑	HSC proliferation ↑, neutrophil recovery ↑, inflammation ↓.^[^ ^79^ ^]^
FR‐WBH	40 °C for 12 h in vitro; 39.5 ± 0.5 °C for 6 h in vivo	WT C57BL/6J, Itga4^R985A/R985A^ C57BL/6J mice	T cells	39.5–40 °C → HSP90, α4 integrins ↑ → T cell adhesion ↑; FAK, RhoA GTPase, VCAM‐1, MAdCAM‐1, ICAM‐1, CCR7, S1PR1 ↑	T cell adhesion ↑, migration ↑, and immune surveillance ↑.^[^ ^74^ ^]^
In vitro fever temperature exposure	38–40 °C; 72 h at the specified temperatures and 48 h at 37 °C	Mice, mouse T cell, and human peripheral blood mononuclear cells	CD4^+^ T cells, DCs, B cells, γδ T cells	39 °C → TRPV1, TRPV4 ↑ → Notch1 → Hes1, Hey1 ↑ → Th2 differentiation ↑ → GATA3 ↑, Tbet ↓; IL‐12 ↑ (DCs) → Th2 differentiation ↓;	Th2 differentiation (w/o APC) ↑, Th2 block (w/APC) ↑.^[^ ^11^ ^]^
In vitro fever temperature exposure	38.5–39.5 °C; 39.5 °C for 3–4 days in vitro; peak fever occurs ≈24 h later.	Autoimmune encephalomyelitis model in mice; mouse cell	CD4^+^ T cells, DCs	38.5–39.5 °C → HSP70/HSP90 ↑ → SMAD4 SUMOylation ↑, STAT3 ↑, RORγt ↑ → Th17 differentiation ↑, IL17 ↑; CD25, FOXP3 ↓	Th17 differentiation ↑, Th1/Treg ↓, neuroinflammation ↑.^[^ ^80^ ^]^
FR‐WBH	39 °C; 39 °C for 24 h in vitro and 8 h in vivo	Mice; mouse cell	CD8^+^ T cells, DCs	**39 °C →** CD8^+^ T activation ↑ **→ IFN‐γ, CD69, CD98, CD25 ↑**	CD8^+^ T activation ↑, antitumor response ↑.^[^ ^12^ ^]^
In vitro fever temperature exposure	39 °C for 24 h	mouse cell	Macrophage	**39 °C → IL‐10, TNF‐α**, **Arg‐1**, **CD163 ↑ →** M2b ↑	M2b‐like phenotype (anti‐inflammatory ↑, pro‐inflammatory ↓).^[^ ^61^ ^]^
Bacterial infection	16–26 °C; 1–8 days post‐infection (2–3 °C rise in body temperature)	True bony fish models	Immune leucocyte, central nervous system (CNS) cells	26 °C → IL‐1β, TNF‐α, IL‐6, HSP70, HSP90 ↑→ Leukocyte recruitment ↑	Pathogen clearance ↑, tissue repair ↑, inflammatory control ↑.^[^ ^81^ ^]^
Bacterial infection	Behavioural fever: 34 °C for 1–5 days; Hyperthermia experiments: 30, 32, or 34 °C for 3 days and 34 °C for 12–60 h	Nile tilapia	CD3^+^ T cells, HEK 293T cells, OmB cells (tilapia B cell line)	34 °C → HSP70‐ERK1/2 ↑, PGE2‐COX‐2 ↑ → Th1/Th2 differentiation ↑→ IFN‐γ, IL‐10, TGF‐β ↑; FOXP3, CTLA‐4, PD‐L1 ↑; Caspase‐8/3 ↓ → T‐cell survival ↑;	Th1/Th2 differentiation ↑, immunosuppression ↑, T‐cell survival ↑.^[^ ^67^ ^]^
In vitro fever temperature exposure	39 °C; 3–4 day in vitro; 4 days in vivo	Mice, mouse T cell, human T cell	CD4^+^ T cells (e.g., TH0, TH1, TH17, iTreg), CD8^+^ T cells	**39 °C → ETC1 ↓,** MitoROS **↑ → γH2AX**, Glut1 **↑→** P53, STING, cGAS, HSP70 **↑ → IFN‐γ, IL‐17A ↑**	CD4^+^ T proliferation ↑, iTreg inhibition **↓**, and metabolic adaptation ↑.^[^ ^62^ ^]^

**Abbreviation**: Fc Receptor, FcR; Complement component 3b, C3b; High Endothelial Venule, HEV; Glucose Regulated Protein 170, GRP 170; Granulocyte, macrophage colony stimulating factor, GM‐CSF; Chemokine (C, C motif) ligand 1/19/12, CCL1/19/12; Interferon‐γ, IFN‐γ; Human Leukocyte Antigen‐DR isotype, HLA‐DR; The Cluster of differentiation 80/86/83/1a/14/25/69/98/163, CD80/86/83/1a/14/25/69/98/163; Natural killer group 2 member D, NKG2D; MHC class I polypeptide, related sequence A Gene, MICA; Granulocyte colony stimulating factor, G‐CSF; C, X, C chemokine receptor type 4, CXCR4; Focal adhesion kinase, FAK; The Rho family of GTPases, RhoA GTPase; Vascular Cell Adhesion Molecule 1, VCAM‐1; Mucosal Addressin Cell Adhesion Molecule 1, MAdCAM‐1; Intercellular Adhesion Molecule 1, ICAM‐1; C, C Chemokine Receptor Type 7, CCR7; Sphingosine, 1, Phosphate Receptor 1, S1PR1; GATA Binding Protein 3, GATA3; T‐box Expressed in T Cells, Tbet; Transient receptor potential cation channel subfamily V member 1/4, TRPV1/4; Antigen‐Presenting Cell, APC; Hairy and Enhancer of Split 1, Hes1; Hairy/Enhancer, of, Split Related with YRPW Motif 1, Hey1; Notch Homolog 1, Notch1; Forkhead Box P3, FOXP3; cytotoxic T‐lymphocyte‐associated protein 4, CTLA‐4; Programmed Cell Death Ligand 1, PD‐L1; RAR, Related Orphan Receptor Gamma t, RORγt; Mothers Against Decapentaplegic Homolog 4, SMAD4; Signal Transducer and Activator of Transcription 3, STAT3; Inducible Nitric Oxide Synthase, INOS; Arginase‐1, Arg‐1; Transforming Growth Factor Beta, TGF‐β; Phosphorylated Extracellular Signal, Regulated Kinase 1/2, ERK1/2; cysteinyl aspartate‐specific proteinase‐8 and proteinase‐3, caspase‐8/3; Glucose Transporter 1, Glut1; Electron transport chain complex 1, ETC1; Tumor Protein p53, p53; Stimulator of interferon genes, STING; Cyclic GMP, AMP Synthase, cGAS; Phosphorylated Histone H2AX, γH2AX; Mitochondrial Reactive Oxygen Species, MitoROS.

#### 2.2.1 Innate Immunity

Fever strengthens innate immunity through a cascade of molecular and cellular mechanisms.^[^ ^21^, ^67^ ^]^ At the molecular level, fever activates heat shock factor 1 (HSF1), which upregulates HSPs (such as HSP70 and HSP110) to bolster cellular stress resistance and immune adaptation.^[^ ^63^, ^68^ ^]^ Moreover, activated HSF1 binds to heat‐shock elements in the non‐classical chemotactic heat shock protein CXCL8 (IL‐8) promoter, driving CXCL8 expression. Increased CXCL8 expression enhances neutrophil release from the bone marrow, accelerates their targeted migration to inflammatory or infected sites, and improves their bactericidal and pathogen clearance capabilities. Above 41.8 °C, however, hyperthermia impairs neutrophil recruitment and function.^[^ ^7^, ^21^, ^63^, ^69^, ^70^ ^]^ Additionally, fever further potentiates NK cell cytotoxicity by inducing MICA expression on tumor cells and clustering NKG2D receptors on NK cells.^[^ ^8^, ^10^, ^21^ ^]^ Fever also upregulates TLR2 and TLR4 on macrophages, enhancing pathogen recognition and triggering the release of pro‐inflammatory mediators, including TNF‐α and HSP70.^[^ ^9^, ^68^, ^71^, ^72^ ^]^ Yet the evidence also indicates that thermal stress dampens cytokine synthesis following macrophage activation.^[^ ^21^ ^]^ Mechanistically, HSF1 represses pro‐inflammatory transcription while blocking NF‐κB promoter binding.^[^ ^82^ ^]^ To DCs, fever strengthens their antigen presentation, fostering robust T‐cell activation and bridging innate and adaptive immune responses.^[^ ^21^, ^70^, ^73^ ^]^ Specifically, 38–41 °C fever upregulates the expression of TLR2/4, major histocompatibility complex (MHC) molecules, and costimulatory molecules on DC surfaces, enhancing their pathogen recognition, phagocytosis, and antigen cross‐presentation capabilities. Moreover, fever also activates the CCR7‐CCL21 axis, which promotes DC migration to draining lymph nodes, where DCs secrete IL‐12 to drive T‐cell polarization and facilitate pre‐assembly of T‐cell signals for enhanced activation.^[^ ^21^ ^]^ Furthermore, DC‐derived IL‐6 cooperates with HSPs to precisely bridge innate and adaptive immunity.

These coordinated effects establish a comprehensive innate immune defense, which is critical for the therapeutic modulation of systemic immunity in EFT.

#### 2.2.2 Adaptive Immunity

Fever significantly enhances adaptive immunity, promoting efficient pathogen clearance and immune memory formation.^[^ ^21^ ^]^ Fever facilitates the migration of bloodborne B cells and T cells to lymph nodes by upregulating chemokine expression and enhancing lymphocyte‐endothelial cell adhesion, thereby enabling efficient interaction with antigen‐presenting cells.^[^ ^19^, ^21^, ^74^, ^75^ ^]^ Similarly, HSP90, in synergy with α4 integrins, enhances T‐cell adhesion and migration under febrile conditions, facilitating trafficking to infection sites.^[^ ^74^ ^]^ The mechanism can also regulate the directed migration of other α4 integrin‐positive immune cells (such as B cells). Additionally, fever facilitates stable T cell synapse formation, optimizing antigen recognition and driving differentiation of T cells into potent effector cells.^[^ ^21^, ^76^, ^77^ ^]^ Studies have shown that fever augments the proliferative and inflammatory functions of CD4^+^ T cells while boosting CD8^+^ cells’ mitochondrial activity, cytokine production, and antitumor efficacy.^[^ ^12^, ^62^ ^]^ At the molecular level, fever‐induced HSP70 translocates to the nucleus, binding ERK1/2 to promote its phosphorylation, which inhibits caspase‐8 and caspase‐3 cleavage, preventing T‐cell apoptosis.^[^ ^67^ ^]^ Collectively, these mechanisms enhance T cell migration, improve synapse formation and function, and facilitate HSP‐mediated survival, together fostering a robust adaptive immune response.

The fever temperature also activates B cells via multiple mechanisms: fever induces TLR9 expression, activating the ERK and NF‐κB pathways to bolster immune responses;^[^ ^83^ ^]^ fever activates HSF to drive B‐cell proliferation and activation, while facilitating antibody secretion and antigen presentation through the formation of B‐cell‐antigen complexes mediated by HSP90;^[^ ^84^, ^85^, ^86^ ^]^ simultaneously, fever promotes B cell survival by exerting anti‐apoptotic effects through the TLR4 pathway, mediated by HSP60, while reducing oxidative stress via Apg‐2, a member of the HSP110 family.^[^ ^87^, ^88^ ^]^ Taken together, these studies supported that the fever‐induced immunogenic effect could be mediated by adaptive immune cell activation.

### 2.3 Fever for Disease Treatment

**Figure 2:**
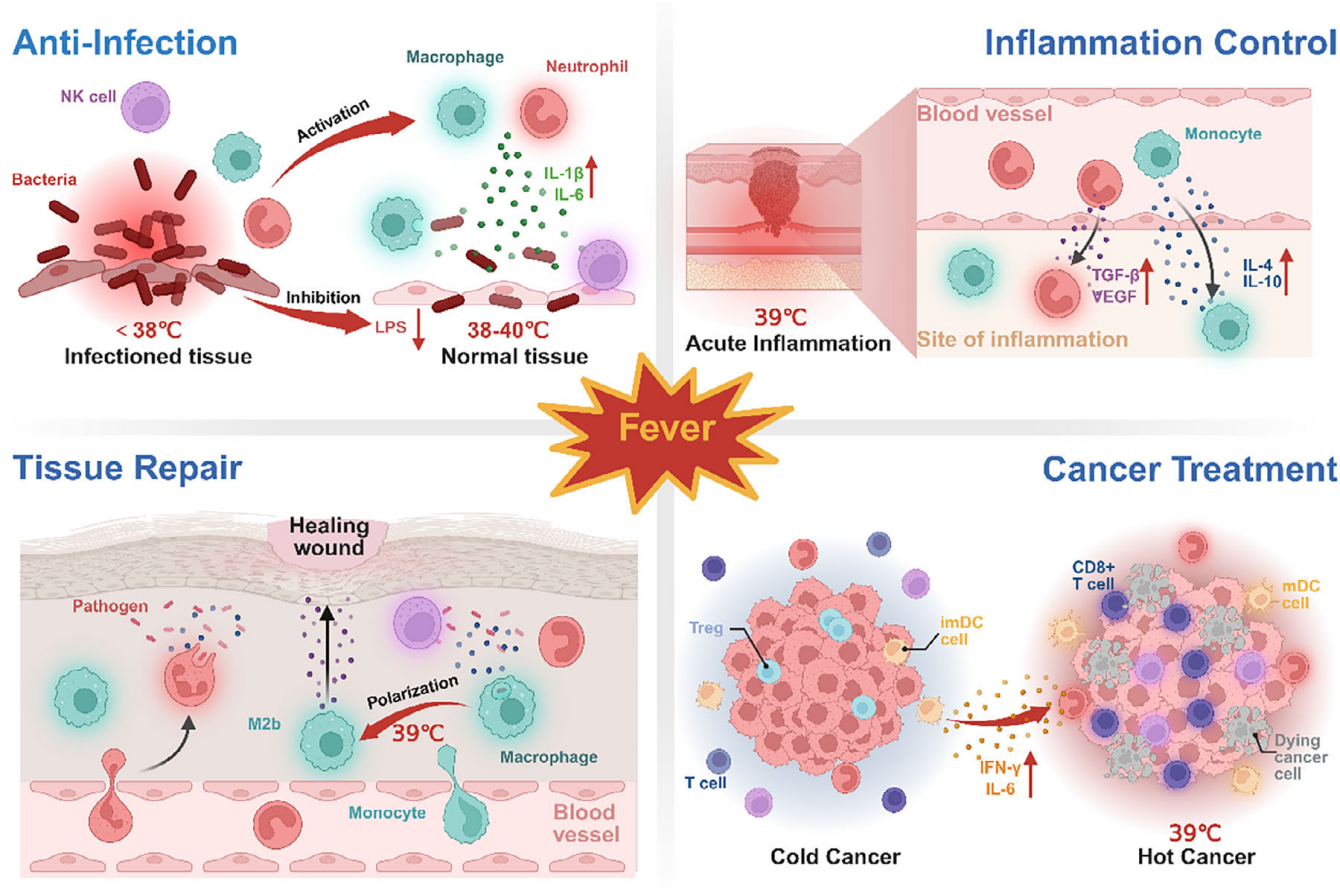
Multifaceted roles of fever in disease management: Anti‐infection, inflammation control, tissue repair, and antitumor immunity. Created in https://BioRender.com.

The immunomodulatory effects of fever confer substantial therapeutic potential across diverse disease contexts, orchestrating robust host defense mechanisms through molecular‐to‐systemic coordination.^[^ ^15^, ^66^, ^81^, ^89^, ^90^ ^]^ However, the literature lacks a comprehensive review of fever's therapeutic applications. This section elucidates the role of fever in combating infections, modulating inflammation, promoting tissue repair, and enhancing cancer treatment outcomes, highlighting its potential for controllable EFT (**Figure** **2**).

#### 2.3.1 Anti‐Infection

Fever serves as a critical defense against infections, exerting antimicrobial effects and improving clinical outcomes. Within physiological range (38–40 °C), fever is a defensive response to infection and is well‐documented to enhance host survival following infection.^[^ ^21^, ^86^, ^91^, ^92^, ^93^ ^]^ Fever inhibits bacterial proliferation (e.g., *Escherichia coli*) by disrupting LPS synthesis while amplifying cytokine release (e.g., IL‐1β, IL‐6) from immune cells to enhance pathogen recognition and clearance.^[^ ^21^, ^94^ ^]^ Similarly, it suppresses 95% of fungal species, with each 1 °C increase between 30–40 °C reducing fungal growth by an additional 6%.^[^ ^95^ ^]^ Clinically, Kushimoto et al. demonstrated that hypothermia in severe sepsis patients (<36 °C) correlates with greater disease severity, reduced adherence to treatment protocols, and elevated mortality risk compared to febrile patients.^[^ ^92^ ^]^ Moreover, suppressing fever may increase infection transmission, underscoring its protective role.^[^ ^96^ ^]^

#### 2.3.2 Inflammation Modulation and Tissue Repair

Fever modulates inflammation and accelerates tissue repair by balancing pro‐inflammatory and anti‐inflammatory responses.^[^ ^21^, ^62^, ^97^ ^]^ In a cold‐blooded vertebrate model, Haddad et al. demonstrated that natural fever and 26 °C mechanical fever (i.e., maintaining a high temperature in the environment through physical means) resulted in more wound healing than that in the 16 °C static temperature group.^[^ ^81^ ^]^ Fever expedites leukocyte recruitment, cytokine modulation, and the expression of anti‐inflammatory and pro‐repair factors, including IL‐4, IL‐10, TGF‐β, and vascular endothelial growth factor (VEGF). Moreover, fever at 39 °C significantly attenuates macrophage‐driven acute inflammation by promoting a shift from pro‐inflammatory M1 to regulatory M2b macrophage phenotypes early in infection.^[^ ^61^ ^]^ These mechanisms, including leukocyte recruitment, cytokine regulation, and M2b polarization, collectively strengthen tissue repair and immune homeostasis.

#### 2.3.3 Cancer Treatment

Fever significantly enhances cancer therapeutic effect by amplifying the immune‐mediated tumor control. Historical observations and extensive case studies, including Rohdenburg's analysis of 302 tumor regression cases, have linked high fever (>39 °C) in post‐incomplete surgical interventions to spontaneous remission in cancers such as childhood leukemia and melanoma.^[^ ^23^, ^98^, ^99^, ^100^, ^101^, ^102^ ^]^ Epidemiological data further reveal a lower cancer incidence among individuals with frequent febrile infections, suggesting fever's protective role against tumorigenesis.^[^ ^14^, ^23^, ^103^, ^104^, ^105^ ^]^ Mechanistically, fever promotes DCs maturation, T‐cell proliferation and differentiation, neutrophil migration, and the secretion of critical cytokines (e.g., IL‐6, interferons) and antibodies.^[^ ^9^, ^13^, ^21^ ^]^ Moreover, fever augments the efficacy of immunotherapies, such as PD‐1 and IL‐2 therapies, with its occurrence during treatment significantly correlating with improved patient survival outcomes.^[^ ^106^, ^107^, ^108^, ^109^ ^]^

Based on the foregoing, fever constitutes a multifaceted mechanism in disease management, orchestrating robust immune responses to enhance host defense. However, prolonged or excessively high fevers (>39 °C) risk adverse outcomes, including tissue damage and metabolic stress.^[^ ^62^, ^110^, ^111^ ^]^ So the precise control of fever parameters (e.g., temperature and duration) is necessary for maximizing the therapeutic effect of fever.

### 2.4 Research Progress and Considerations in Engineered Fever Therapy

EFT is a clinical intervention designed to elevate the patient's core body temperature, amplifying systemic anti‐tumor immune responses and enhancing cancer treatment outcomes.^[^ ^112^ ^]^

**Figure 3:**
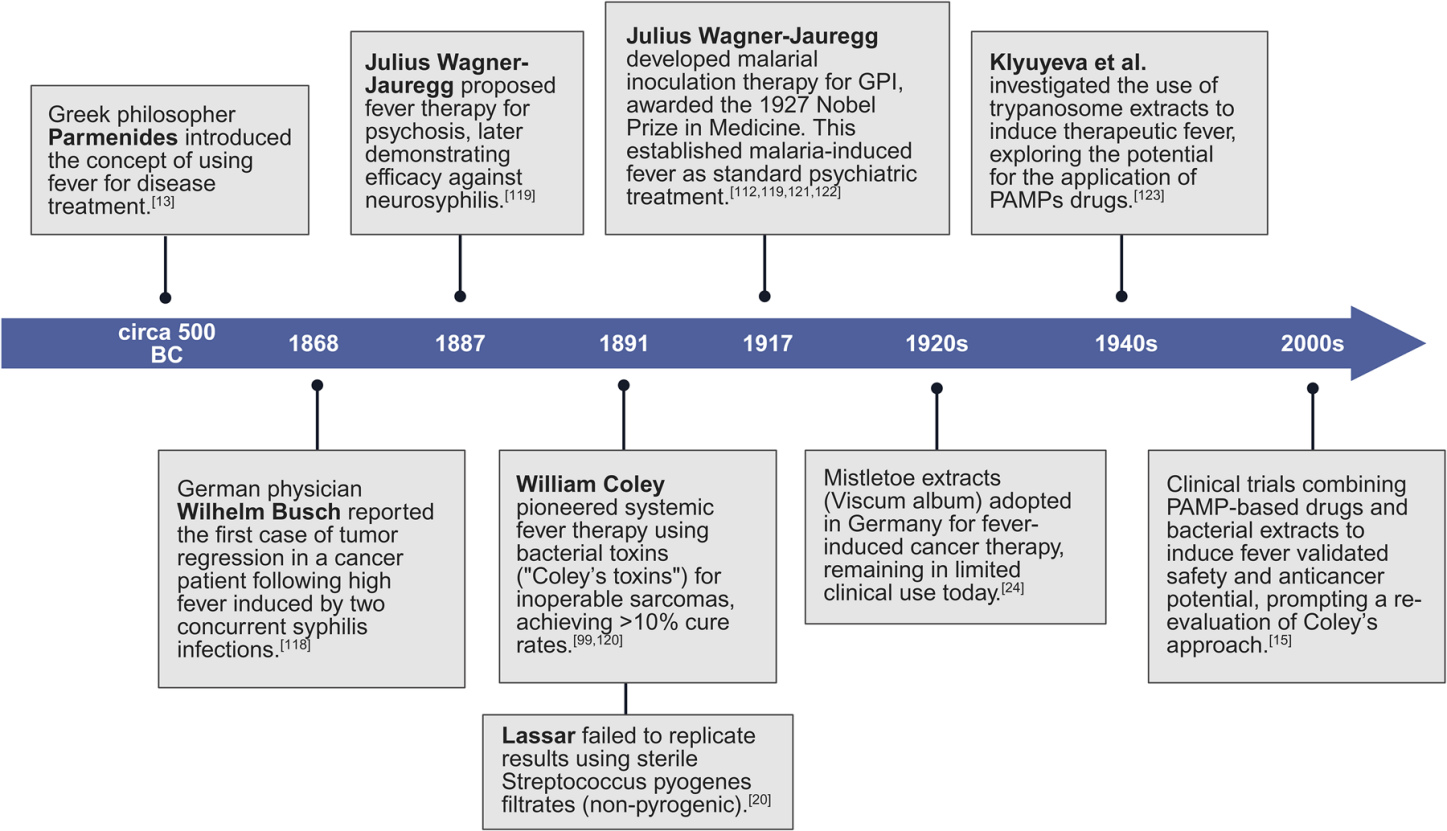
Research progress in engineered fever therapy.^[^ ^13^, ^15^, ^20^, ^24^, ^99^, ^112^, ^118^, ^119^, ^120^, ^121^, ^122^, ^123^ ^]^ Created in https://BioRender.com.

Over the past century, EFT has demonstrated substantial potential in eliciting robust immunological cascades to combat malignancies (**Figure** **3**). By harnessing fever's immunomodulatory effects, EFT offers a novel adjuvant strategy to enhance anti‐tumor immunity, particularly against metastatic lesions.^[^ ^21^ ^]^ For instance, Reuter et al.^[^ ^15^ ^]^ conducted a retrospective Phase I study to assess the safety and feasibility of fever induction using approved drugs and bacterial preparations containing PAMPs in cancer patients for therapeutic hyperthermia. Building on historical precedents such as Coley's toxin, the study enrolled 131 predominantly advanced‐cancer patients who received a total of 523 fever‐induction procedures. Patients were divided into Group A (Se/Ps bacterial extracts; A1 without pretreatment, A2 with pretreatment) and Group B (19 combinations of 7 PAMP‐containing drugs, all pretreated) using dose‐escalation titration. Both agents consistently induced fever (38.5–40 °C) with no serious adverse events across all applications. Pretreatment reduced mild side effects, and prior chemoradiotherapy did not impair the induction of fever. Notably, two patients exhibited clinical improvement, though this was an incidental observation. Similarly, Orange et al.^[^ ^113^ ^]^ reported two cases of patients who declined conventional cancer treatment and achieved sustained tumor remission using high‐dose Viscum album extracts combined with multiple administration routes and fever induction. While these case reports (*n* = 2) indicate potential benefits and demonstrate the feasibility of fever‐based therapies, their uncontrolled and retrospective designs introduce inherent biases and preclude broad generalizability. Therefore, larger randomized controlled trials are imperative to robustly validate the efficacy.^[^ ^114^ ^]^

Fever's therapeutic potential is balanced against associated risks. Prolonged or excessive hyperthermia can induce mitochondrial stress, DNA damage, and apoptosis in critical immune cell subsets, such as Th1 cells, potentially exacerbating oncogenic processes.^[^ ^62^ ^]^ Moreover, conventional EFT, which relies on bacterial extracts or pharmacological agents, suffers from imprecise temperature control, adverse side effects, and associated toxicities that limit its scalability and clinical safety, despite some mitigation from dose titration. To address these challenges, precise control of fever parameters, including temperature (38.5–40 °C) and duration (4–6 h), is essential to optimize therapeutic efficacy while ensuring patient safety. Recent advancements in nanomedicine‐based thermal immunotherapy, characterized by precise spatiotemporal modulation and targeted delivery, may provide critical insights into the development of advanced EFT approaches.^[^ ^26^, ^115^, ^116^, ^117^ ^]^ Specifically, integrating precise nanomedicine approaches with EFT combines fever‐induced systemic immune stimulation and localized thermal immunotherapy, enhancing both safety and therapeutic impact.

## 3 Engineered Thermal Immunotherapy to Mimic the Effects of Natural Fever

Engineered thermal immunotherapy employs advanced photothermal (PTT), magnetothermal (MTT), and nanomedicine‐based techniques to target and ablate malignant cells while eliciting a robust immunological response.^[^ ^26^, ^124^ ^]^ This precision‐driven approach enhances antitumor efficacy by synergizing with immunotherapies, such as immune checkpoint inhibitors, to optimize therapeutic outcomes in precision oncology.^[^ ^25^, ^26^, ^125^, ^228^ ^]^

### 3.1 Thermally Driven Mechanisms of Engineered Thermal Immunotherapy

Engineered thermal immunotherapy leverages precisely controlled hyperthermia to induce immunogenic cell death (ICD) and enhance antitumor immunity, serving as the cornerstone of advanced cancer treatment strategies. At the molecular level, mild hyperthermia, typically within the temperature range of 41–45 °C, induces protein denaturation and aggregation, disrupting critical cellular functions in cancer cells, such as DNA repair function.^[^ ^26^, ^126^ ^]^ This process promotes the release of damage‐associated molecular patterns (DAMPs), including calreticulin, ATP, and high‐mobility group box 1 (HMGB1), which recruit DCs and enhance tumor‐associated antigen presentation to cytotoxic T‐cells, transforming tumors from immunologically “cold” (non‐immunogenic) to “hot” (immunogenic) phenotypes.^[^ ^25^, ^68^, ^127^, ^128^, ^129^ ^]^ At the tissue level, thermal immunotherapy augments tumor microenvironment (TME) vascular perfusion and permeability, facilitating immune cell infiltration, enhancing therapeutic agent delivery, and mitigating TME immunosuppression.^[^ ^130^, ^131^ ^]^ Collectively, these thermally driven mechanisms synergistically amplify antitumor immunity, significantly improving the efficacy of immunotherapeutic strategies.

### 3.2 Nanomedicine‐Based Thermal Immunotherapy

Nanomedicine‐based thermal immunotherapy (NTI) integrates multifunctional nanomaterials with thermal modalities, such as PTT, MTT, and sonothermal therapies, to establish a precision‐driven therapeutic platform for cancer treatment (**Table** **2**).^[^ ^26^, ^132^, ^133^, ^134^ ^]^ Several thermal therapeutic modalities target the activation of HSP70/90, ICD, and DC, aligning with the immune pathways modulated by fever and its associated temperature regulation, highlighting critical immune mechanisms in heat‐based therapies. Among them, PTT (42–50 °C) and radiofrequency therapy (45–55 °C) induce ICD, releasing DAMPs to activate DCs, while MTT (42–46 °C) enhances HSP70/90‐mediated antigen presentation.^[^ ^117^, ^135^, ^136^, ^139^ ^]^ Additionally, PTT and MTT exhibit significant clinical potential for melanoma and brain tumors, synergizing with immune checkpoint inhibitors.

**Table 2:** The main thermal immunotherapy modalities based on nanomedicine.

Modality	Therapy temperature	Immunological mechanism and effect	Pros and Cons
PTT^[^ ^25^, ^117^, ^135^ ^]^	42–50 °C	Thermal induces ICD in tumor cells, releasing DAMPs, activating DCs for tumor antigen presentation to T cells, and reducing immunosuppressive cells (Tregs, M2 macrophages) while increasing tumor‐infiltrating lymphocytes.	Pros: high spatiotemporal precision and high photothermal efficiency for synergistic therapeutic potential; Cons: uneven temperature control, weak immune response, and limited clinical translation.
MTT^[^ ^117^, ^131^, ^136^ ^]^	42–46 °C	Thermal triggers tumor cell expression of HSP70/90, boosts antigen presentation efficiency, and stimulates IL‐6 and TNF‐α secretion to activate innate immunity and enhance immune recognition of tumor cells.	Pros: deep tissue applicability, non‐invasive, and simultaneous use for magnetic resonance imaging (MRI) imaging‐guided treatments; Cons: uneven heat distribution and prolonged retention of magnetic particles may trigger inflammation or fibrosis.
Sonothermal therapy^[^ ^137^, ^138^ ^]^	42–50 °C	Thermal promotes tumor antigen release and enhances immune cell infiltration, synergizing with thermotherapy to amplify anti‐tumor immune activation.	Pros: excellent deep tissue penetration without ionizing radiation for precise focusing; Cons: ultrasound parameters need to be precisely controlled to avoid normal tissue damage.
Radiofrequency thermal therapy^[^ ^139^, ^140^ ^]^	45–55 °C	Thermal ablation induces ICD and enhances dendritic cell maturation.	Pros: non‐invasive, suitable for multi‐site tumors, and some materials (e.g., gold) can be used for imaging; Cons: high‐power equipment is required and may cause overheating of surrounding tissues.
Microwave thermal therapy^[^ ^141^, ^142^, ^143^ ^]^	41–43 °C	Thermal with microwaves disrupts the tumor stromal barrier, facilitating immune checkpoint inhibitor penetration to improve efficacy of checkpoint blockade and immune cell access to the TME.	Pros: rapid heating, suitable for large‐volume tumors, and some materials have MRI compatibility. Cons: microwave scattering may affect focusing.

Leveraging nanoparticles with precise targeting capabilities, spatiotemporal tunability, and high thermal conductivity, NTI enables finely controlled modulation of immunogenic thermal effects at the molecular, cellular, and tissue levels.^[^ ^26^, ^144^, ^145^, ^146^, ^147^ ^]^ These capabilities enhance localized thermal interventions, reconfigure the tumor‐suppressive TME, and amplify the efficacy of complementary immunotherapies, such as immune checkpoint inhibitors.^[^ ^25^, ^131^, ^148^, ^149^, ^150^ ^]^ This section provides an overview of key NTI approaches—photothermal and magnetothermal therapies—detailing their strategies for thermal immunomodulation. For a comprehensive analysis of additional NTI modalities, readers can refer to specialized reviews.^[^ ^26^, ^137^ ^]^

#### 3.2.1 Photothermal Immunotherapy

PTT employs photothermal agents (PTAs) to convert light into localized heat, effectively inducing tumor cell ablation while simultaneously eliciting anti‐tumor immune responses through heat‐mediated cellular stress.^[^ ^151^, ^152^, ^153^ ^]^ Owing to its high selectivity and minimally invasive nature, PTT is widely recognized as a promising strategy for precision cancer treatment. The efficacy of PTT hinges critically on the thermal performance of PTAs like gold nanoparticles (Au NPs),^[^ ^154^, ^155^ ^]^ polydopamine (PDA),^[^ ^156^, ^157^ ^]^ and molybdenum disulfide nanoparticles (MoS_2_ NPs).^[^ ^150^ ^]^ Each PTA exhibits unique physicochemical properties and photothermal conversion mechanisms, enabling tailored thermal modulation.^[^ ^135^, ^158^, ^159^ ^]^ Moreover, integration with immune checkpoint inhibitors (e.g., anti‐CTLA‐4 or anti‐PD‐L1 antibodies) amplifies thermally induced immune activation and enhances T‐cell responses.^[^ ^148^, ^160^, ^161^ ^]^ Several exemplary studies illustrate the pivotal role of thermal effects and associated immune markers in enhancing PTT‐based immunotherapy.

**Figure 4:**
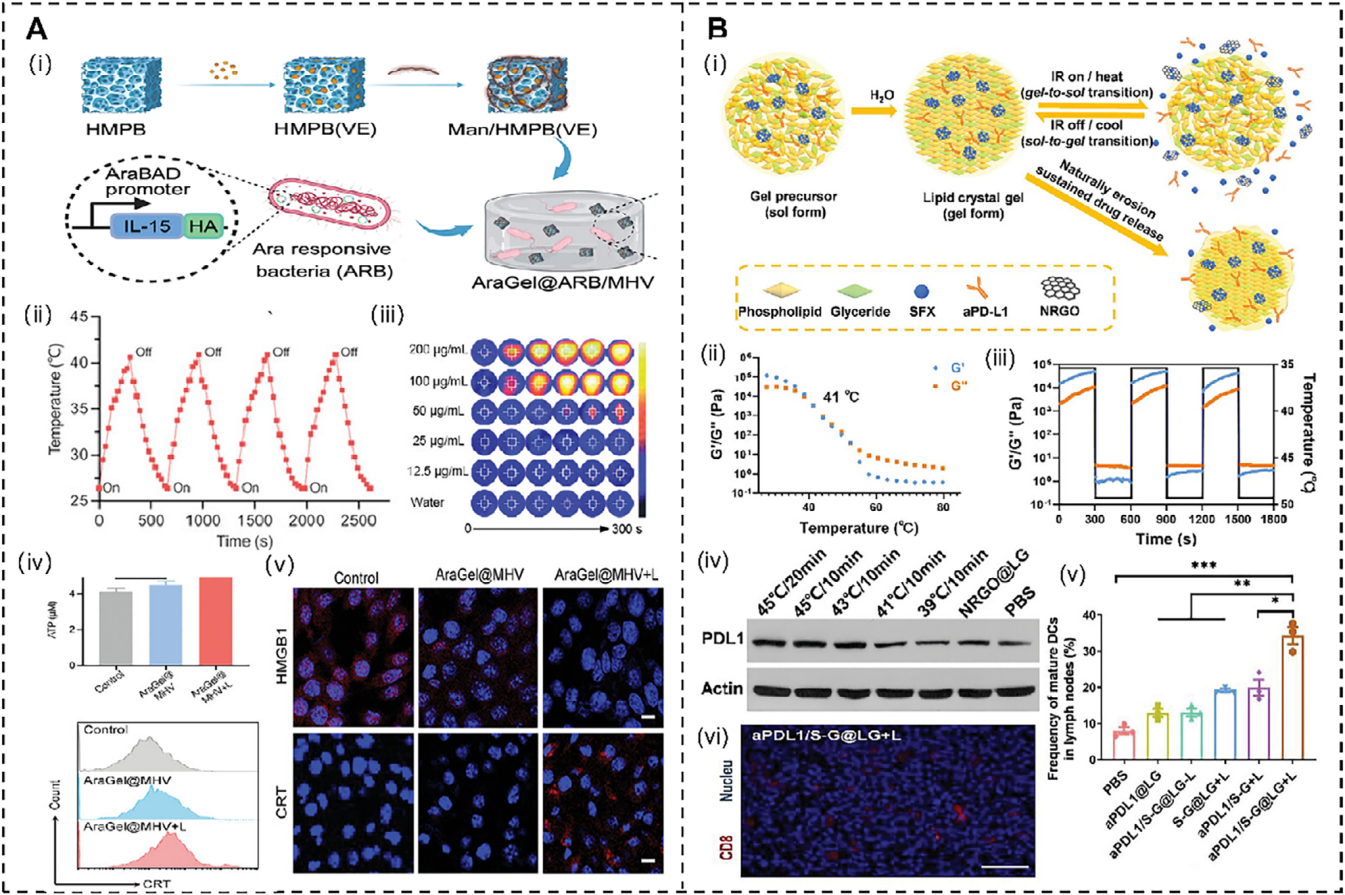
A) Schematic illustration of AraGel@ARB/MHV (AAM) system for TDLN remolding, self‐adjuvanting bacteria, and immunogenic cell death. Reproduced with permission.^[^ ^162^ ^]^ Copyright 2024, Wiley‐VCH GmbH. B) Schematic illustration of combination immunotherapy based on reshaping of tumor microenvironment by mild photothermal therapy along with exosome inhibition to potentiate immune checkpoint blockade therapy. Reproduced with permission.^[^ ^163^ ^]^ Copyright 2024, Wiley‐VCH GmbH.

The thermal effects of PTT significantly enhance the efficacy of immunomodulatory agents by inducing ICD and improving vascular perfusion in TME. Pan et al.^[^ ^162^ ^]^ developed a self‐adjuvanting hydrogel platform (AraGel@ARB/MHV, AAM) combining mild PTT (≈45 °C), SH2‐containing protein tyrosine phosphatase‐1 (SHP1) checkpoint inhibition, and IL‐15 delivery to target tumor‐draining lymph nodes (TDLNs), as shown in **Figure** **4**A. The thermal effect, mediated by mannose‐modified hollow mesoporous Prussian blue nanoparticles (Man/HMPB), induces ICD, releasing tumor antigens and DAMPs (e.g., calreticulin, HMGB1). These thermally triggered markers enhance SHP1 inhibition and IL‐15 activity by recruiting and activating APCs, upregulating co‐stimulatory markers (CD80^+^, CD86^+^) on DCs, and boosting T‐cell priming and activation in the TME. Similarly, Chen et al.^[^ ^163^ ^]^ engineered a photothermal‐responsive nano‐complex (NRGO@LG), integrating mild PTT (45 °C), anti‐PD‐L1 antibodies, and exosome inhibitor sulfisoxazole, as shown in Figure 4B. The strategy leverages controllable hyperthermia to remodel the immunosuppressive “cold” TME, achieving multi‐level thermal immunomodulation. At the molecular level, PTT‐induced ICD releases DAMPs, thereby amplifying the efficacy of anti‐PD‐L1, despite the upregulation of immunosuppressive tumor exosomes and PD‐L1. At the cellular level, hyperthermia enhances DC activation (marked by elevated expression of CD80, CD86, and CCR7) and increases cytotoxic CD8^+^ T‐cell populations (Gzmb^+^), while reducing T‐cell exhaustion. At the tissue level, thermally improved vascular perfusion further facilitates greater immune cell infiltration into the TME, enhancing anti‐PD‐L1‐mediated immunity. These thermal‐driven effects directly amplify the immunomodulatory agents’ ability to drive robust anti‐tumor responses.

**Figure 5:**
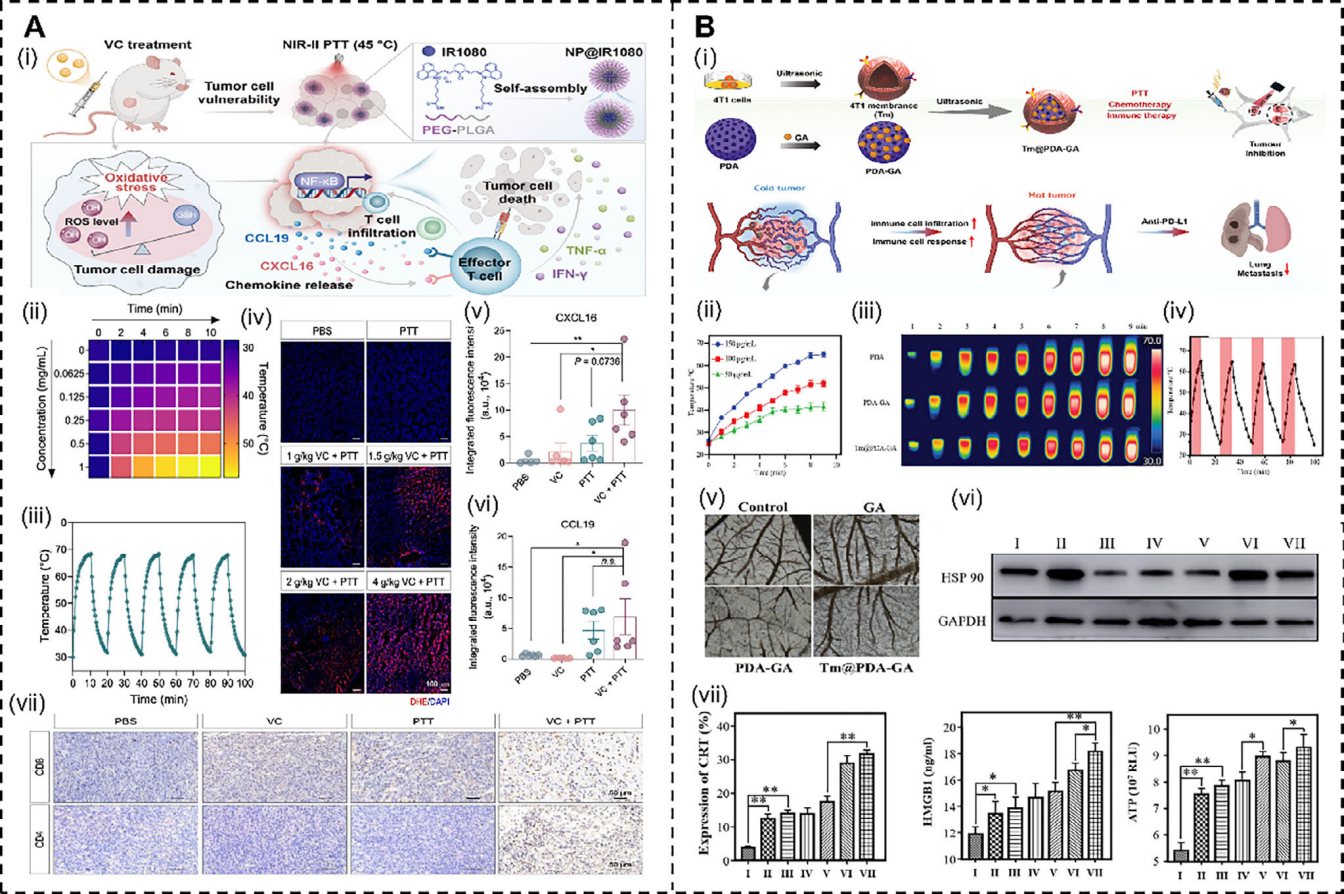
A) Schematic illustration of the synergistic antitumor effects of high‐dose VC and NIR‐II PTT mediated by NPs@IR1080. Reproduced with permission.^[^ ^164^ ^]^ Copyright 2025, American Chemical Society. B) Design and specific features of the drug delivery system Tm@PDA‐GA. Reproduced with permission.^[^ ^165^ ^]^ Copyright 2024, Wiley‐VCH GmbH.

Thermal effects similarly enhance vitamin C (VC) and vascular‐modulating drugs by amplifying immune markers and improving TME dynamics.^[^ ^116^, ^126^, ^144^, ^164^, ^165^ ^]^ As displayed in **Figure** **5**A, Deng et al.^[^ ^164^ ^]^ demonstrated that near‐infrared‐II (NIR‐II) PTT, augmented by high‐dose VC, drives thermoregulatory immunomodulation through pronounced thermal effects. The localized heat from PTT synergizes with VC‐induced reactive oxygen species (ROS) production, depleting glutathione and disrupting mitochondrial function, thereby amplifying heat‐activated NF‐κB signaling to upregulate the expression of CXCL16 and CCL19. These thermally induced chemokines enhance the infiltration of CXCR6^+^ CD8^+^ and CD4^+^ T cells, exhibiting effector and central memory phenotypes, with support from co‐recruited populations of macrophages and neutrophils. Furthermore, PTT's thermal effects increase tumor vascular permeability and perfusion, facilitating immune cell infiltration and CXCR6^+^ and CCR7^+^ T‐cell recruitment, which collectively drive potent anti‐tumor immunity. In another study, Lan et al.^[^ ^165^ ^]^ constructed a Tm@PDA‐GA nanoplatform to synergize PTT with vascular normalization to enhance immunotherapy for triple‐negative breast cancer (Figure 5B). Among them, PTT‐induced ICD and gambogic acid‐mediated HSP90 inhibition enhance thermal sensitivity, while HIF‐1α/VEGF pathway‐driven vascular normalization promotes subsequent immune infiltration.

For more detailed information, one can refer to several excellent reviews for a more comprehensive understanding of the therapy.^[^ ^166^ ^]^

#### 3.2.2 Magnetothermal Immunotherapy

MTT represents another promising modality in cancer treatment, leveraging its exceptional deep‐tissue penetration to deliver targeted hyperthermia. By applying alternating magnetic fields (AMFs), MTT activates magnetic nanomaterials (e.g., superparamagnetic iron oxide nanoparticles) to generate localized thermal effects that induce tumor cell ablation and stimulate immune responses.^[^ ^167^, ^168^, ^169^ ^]^ Recent advancements have focused on optimizing nanomaterial physicochemical properties—size, morphology, composition, and magnetic anisotropy—to enhance thermal conversion efficiency and therapeutic efficacy.^[^ ^81^, ^167^ ^]^ Similarly, integrating MTT with other therapeutic modalities (e.g., immune checkpoint inhibitors and ferroptosis) has demonstrated substantial synergy in improving thermal and antitumor efficacy, particularly in treating metastatic tumors.^[^ ^136^, ^160^, ^170^ ^]^

**Figure 6:**
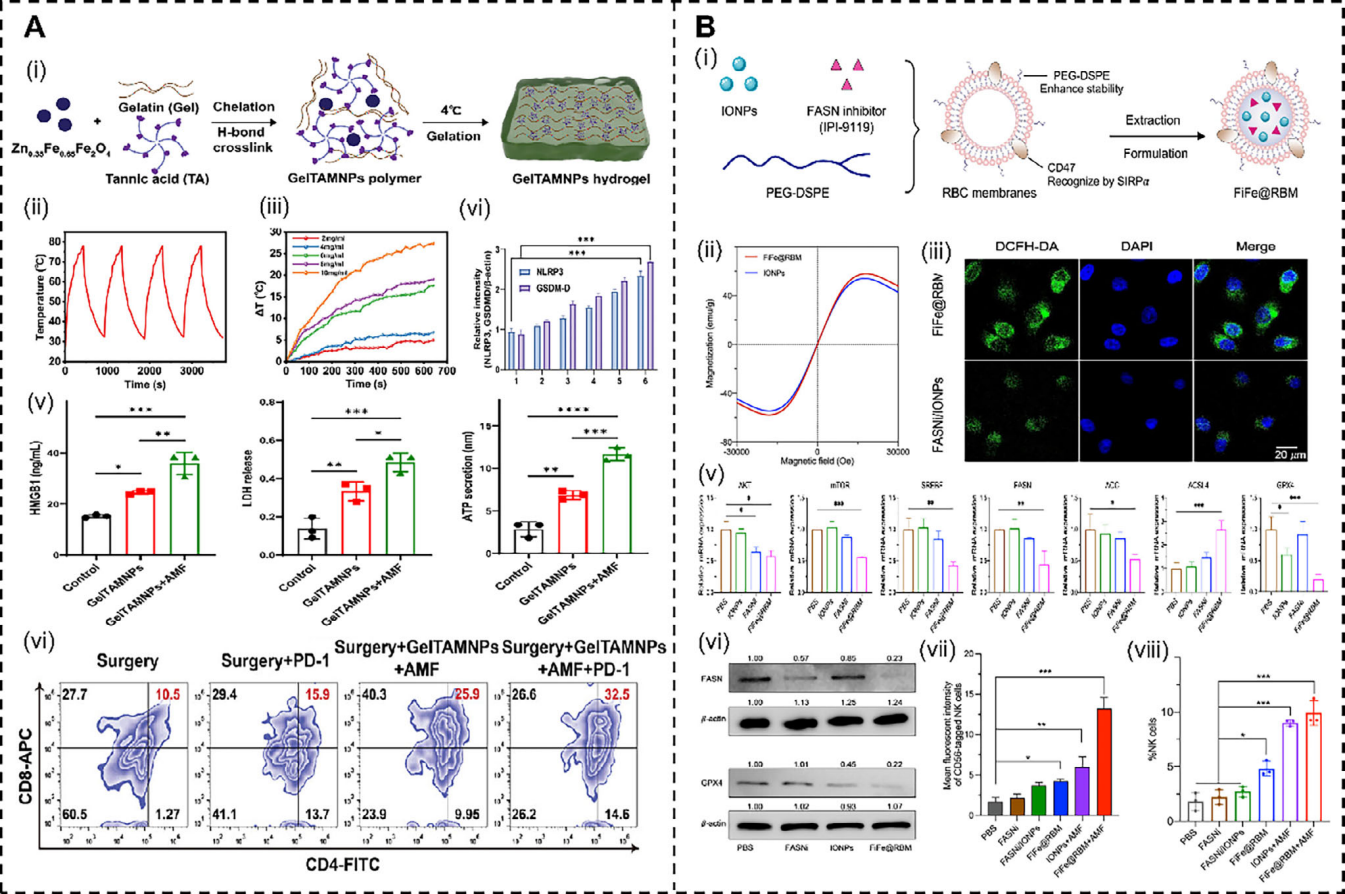
A) Mechanism of action and preparation of the magnetic thermal‐responsive smart hydrogel (GelTA‐(Zn0.35Fe0.65) Fe_2_O_4_). With the combination of PD‐1 checkpoint blockade, an efficient abscopal effect, leads to tumor recurrence and metastasis regression. Reproduced with permission. Reproduced with permission.^[^ ^171^ ^]^ Copyright 2024, Wiley‐VCH GmbH. B) Schematic illustration of engineering FiFe@RBM and the mechanism of rewiring lipid metabolism to enhance ferroptosis and synergize with magnetic hyperthermia for effective treatment of CRPC and inhibiting liver metastasis through activated NK cells. Reproduced with permission.^[^ ^172^ ^]^ Copyright 2025, American Chemical Society.

For instance, Wang's group^[^ ^171^ ^]^ designed a gelatin‐tannic acid gel (GelTA) crosslinked bimetallic magneto‐thermally reactive hydrogel (GelTAMNPs) (**Figure** **6**A). Under AMF‐induced hyperthermia, GelTAMNPs mediate thermoregulatory immunomodulation to suppress post‐surgical tumor recurrence. The localized heat triggers pyroptosis, releasing DAMPs (IL‐18, IL‐1β, and tumor antigens), which enhance tumor immunogenicity. Thermally induced pyroptosis further promotes DC maturation and antigen presentation, recruiting CTLs and amplifying immune responses. Concurrently, hyperthermia facilitates lymph node delivery of tumor antigens and cytokines, acting as a “relay baton” to stimulate systemic immunity. Combined with PD‐1 blockade, thermally driven immunomodulation significantly enhances anti‐tumor efficacy, showcasing a synergistic interplay between heat‐induced pyroptosis and systemic immune activation. Cheng et al.^[^ ^172^ ^]^ developed biomimetic nanoparticles (FiFe@RBM), illustrated in Figure 6B, encapsulating fatty acid synthase inhibitors with iron oxide nanoparticles for depot‐resistant prostate cancer therapy. In this system, thermal effects promote iron ion release, synergizing with the Fenton reaction to enhance ROS production, inhibit the protein kinase B‐mammalian target of rapamycin (AKT‐mTOR) pathway, and downregulate glutathione peroxidase 4 expression. This reprogramming of lipid metabolism, evidenced by increased polyunsaturated fatty acid‐phospholipid accumulation, augments ferroptosis. Concurrently, hyperthermia‐induced mitochondrial damage and immunogenic cell death release DAMPs, activating and recruiting a high proportion of NK cells to infiltrate tumor tissue, significantly suppressing liver metastases while ablating primary tumors.

Overall, PTT and MTT emphasize the immune effects in NTI, including HSP‐mediated antigen presentation and cytokine release. They link innate and adaptive immunity via thermally induced ICD, DAMPs, and chemokines. These mechanisms provide a foundation for controllable EFT.

## 4 Controllable Engineering Fever Therapy

However, both NTI and EFT face critical limitations that hinder their standalone clinical efficacy, underscoring the urgent need for their integration. Among them, EFT faces challenges in achieving precise systemic control and ensuring safety. The limitations of NTI include i) the requirement for sustained high temperatures (>50 °C) to induce tumor cell apoptosis, which risks collateral damage to adjacent healthy tissues; ii) the potential to exacerbate immune resistance within the tumor's immunosuppressive microenvironment; and iii) the localized nature of NTI's thermal immune response, which fails to address distant metastatic lesions effectively.^[^ ^26^, ^145^, ^173^, ^174^, ^175^ ^]^ Furthermore, exogenous temperature manipulation induces physiological stress and cannot replicate the host's intrinsic thermoregulatory mechanisms activated during natural fever.^[^ ^21^ ^]^ Engineered thermal immunotherapy alone is also insufficient to stimulate cytokine production and cytoprotective gene expression in the CNS.^[^ ^81^ ^]^

To overcome complementary limitations of NTI and EFT, integrating NTI with EFT offers a transformative hybrid strategy that forms the core of controllable EFT, enhancing the overall therapeutic efficacy of thermal immunotherapy. By combining EFT's systemic immune activation with NTI's precision‐targeted hyperthermia, their individual shortcomings can be synergistically addressed. For instance, NTI's nanotechnology can enhance EFT's controllability, reducing risks such as cytokine storms or cardiovascular strain. While EFT's systemic effects amplify NTI's localized immune responses to achieve broader anti‐tumor immunity. The mechanisms, immune scope, advantages, and limitations of EFT, NTI, and their integration are summarized in **Table** **3**, facilitating technological optimization and effective clinical application.

**Table 3:** Comparison of EFT, NTI, and controllable EFT.

Aspects	EFT	NTI	Controllable EFT
Definition	Utilizes external stimuli (e.g., PAMPs, bacterial agents) to elevate core body temperature and induce systemic immune responses.^[^ ^112^, ^113^ ^]^	Employs photothermal, magnetothermal, or nanomedicine techniques to deliver localized hyperthermia (40–45 °C) to the tumor.^[^ ^26^, ^124^ ^]^	Combines EFT's systemic immune activation with NTI's targeted hyperthermia to enhance therapeutic outcomes.
Immune scope	Systemic, activates broad innate and adaptive immunity, including DC, NK, T, and B cells.^[^ ^21^ ^]^	Localized, primarily enhances tumor‐specific immune responses (e.g., DC and T cell activation).^[^ ^25^, ^125^ ^]^	Systemic and localized, it amplifies tumor‐specific and whole‐body immunity.
Advantages	Overcomes tumor immunosuppressive microenvironment; promotes whole‐body anti‐tumor immunity.^[^ ^9^, ^21^ ^]^	Precise tumor targeting; minimizes off‐target effects; synergizes with checkpoint inhibitors.^[^ ^26^, ^144^, ^148^ ^]^	Combines systemic immunity with precise targeting; enhances efficacy and safety through controlled modulation.
Limitations	Risk of systemic toxicity (e.g., cytokine storms, cardiovascular strain); uncontrolled; limited clinical data.^[^ ^15^, ^23^, ^24^, ^113^ ^]^	Risk of damaging healthy tissue; limited systemic immune activation; potential immune resistance.^[^ ^26^, ^81^, ^145^, ^173^, ^174^, ^175^ ^]^	The lack of feasible strategies necessitated further exploration of the combined strategies.
Clinical applicability	Emerging applications in cancers with high immunosuppressive microenvironment; potential in combination with checkpoint inhibitors or chimeric antigen receptor T‐Cell (CAR‐T) therapies; have promise in systemic immune activation.	Established in localized tumor treatments; trials for combination with checkpoint inhibitors and chemotherapy.^[^ ^25^, ^26^, ^125^, ^228^ ^]^	Promising for advanced cancers; leverages strengths of both approaches; needs clinical validation.

Unlike traditional biologically induced fever, which suffers from inherent unpredictability, controllable EFT employs physical and bioengineering strategies to achieve precise fever modulation through three key innovations: i) controllable fever induction, enabling precise regulation of input parameters and dynamic process control to mimic physiological fever patterns; ii) quantitative reprogramming of immune cells, leveraging precisely modulated thermal stimuli to induce targeted immune pathway activation; and iii) enhanced safety through controlled fever dynamics, with optimized time and temperature profiles to minimize damage.

Although current research on controllable EFT remains limited, recent advances in NTI demonstrate significant potential in modulating systemic fever‐associated immune responses, paving the way for integrated EFT‐NTI strategies. Our prior work established that photothermal CpG nanotherapeutics (PCN), integrating CpG oligodeoxynucleotides, gold nanorods, and ovalbumen, induce a controlled fever‐like temperature of 43 °C within the tumor microenvironment under near‐infrared irradiation, effectively mimicking systemic fever dynamics.^[^ ^107^ ^]^ High‐throughput gene profiling revealed modulation of nine immune‐related genes, including upregulation of CCL8 and C‐type lectin domain family 4 member E gene for immune cell recruitment and downregulation of immunosuppressive genes (synuclein gamma, indoleamine 2,3‐dioxygenase 2, and colony‐stimulating factor). In 4T1 tumor‐bearing mice, PCN significantly elevated serum levels of IL‐6, IL‐12, and IL‐1β, comparable to LPS‐induced fever, while promoting dendritic cell maturation and robust CD8^+^ T cell infiltration. This fever‐inspired strategy achieved marked tumor suppression, complete regression in select cases, and prolonged survival, underscoring PCN's capacity to regulate systemic immune indicators and establish a framework for controllable EFT. Complementarily, Zhang et al. developed hyaluronic acid‐modified PAMA nanocarriers (HPDS NPs) co‐delivering 2,4‐dinitrophenol (DNP) and syrosingopine (Syro), a mitochondrial uncoupler and MCT‐4 inhibitor, respectively.^[^ ^176^ ^]^ Among them, DNP can convert the electrochemical potential energy of mitochondria into heat to achieve self‐heating of cancer cells. It triggers ICD, releasing tumor‐associated antigens and activating dendritic cells without external devices. Moreover, Syro can inhibit the efflux of lactic acid and work synergistically with DNP to enhance the therapeutic effect on endogenous high fever and suppress immunosuppression and thermal resistance. By integrating spontaneous hyperthermia with immune‐metabolic cross‐regulation, HPDS NPs offer a novel paradigm for controllable EFT.

### 4.1 Central Pattern‐Induced

**Figure 7:**
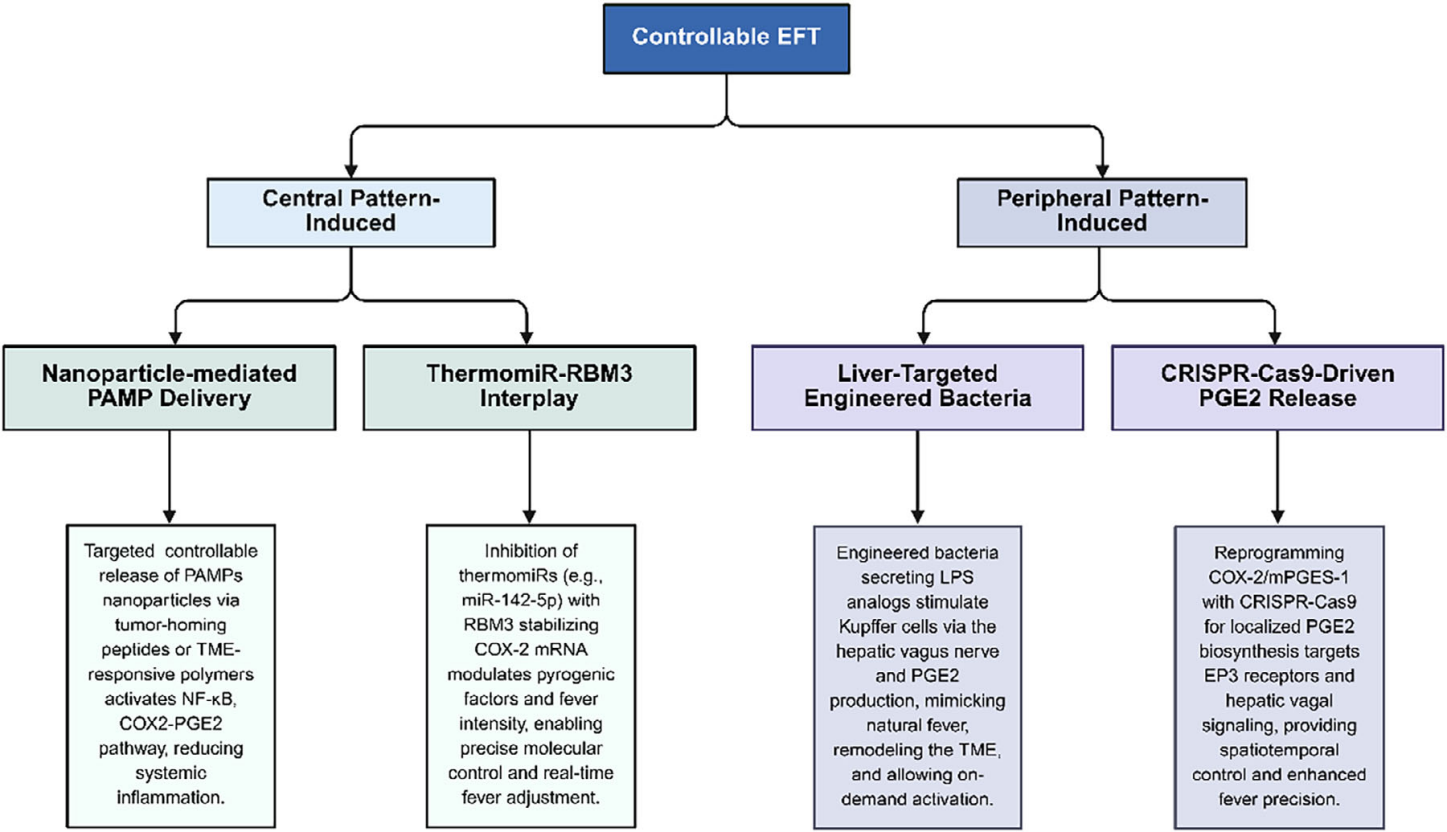
Controllable EFT: central pattern‐induced and peripheral pattern‐induced. Created in https://BioRender.com.

To advance the development of controllable EFT, several innovative regulatory strategies are proposed, encompassing both central and peripheral immune activation mechanisms (**Figure** **7**).

The central pattern of fever induction hinges on peripheral EPs, such as IL‐1β and TNF‐α, which traverse the bloodstream to activate the COX2‐PGE2 axis in brain endothelial cells. Subsequently, the hypothalamic thermoregulatory center is stimulated to elevate body temperature. Nanoparticles provide a robust platform for remotely modulating fever‐associated signaling pathways—namely NF‐κB, COX2, and TRP—in immune cells like KCs and DCs.^[^ ^21^, ^31^, ^32^, ^33^, ^177^, ^178^, ^179^, ^180^ ^]^ Although prior studies have leveraged these pathways to modulate inflammation and reshape the immune microenvironment,^[^ ^181^, ^182^, ^183^ ^]^ temperature dynamics have been underexplored, underscoring the need to prioritize these pathways as regulators of fever. For example, enhancing spatial specificity in fever induced by PAMPs and other broad‐spectrum immune activators.^[^ ^15^, ^22^ ^]^ Nanoparticle‐based delivery systems offer a promising solution, enabling targeted delivery and controlled release of PAMPs through surface modifications such as tumor‐homing peptides or TME‐responsive polymers (e.g., pH‐ or enzyme‐responsive polymers).^[^ ^184^, ^185^ ^]^ These modifications focus on the activation of immune cells within the TME and achieve a progressive transition from local “hot” (inducing targeted immune activation) to systemic fever (promoting broader anti‐tumor immunity), thereby minimizing the risk of systemic inflammation.

Moreover, modulation of natural fever's negative feedback mechanisms offers a compelling strategy for controllable EFT.^[^ ^186^ ^]^ ThermomiRs, such as miR‐142‐5p^[^ ^187^, ^188^, ^189^ ^]^ and miR‐143,^[^ ^34^, ^190^, ^191^ ^]^ serve as molecular switches. Their pharmacological inhibition (e.g., via antagonist oligonucleotides in THP‐1 cells) upregulates pyrogenic factors independently of external heat, thereby ensuring precise control of molecular inputs.^[^ ^186^ ^]^ The cold shock protein RBM3 further enhances controllability by suppressing thermomiR expression while stabilizing COX‐2 mRNA, amplifying pyrogenic output.^[^ ^186^, ^192^ ^]^ The thermomiR‐RBM3 interplay enables process control, dynamically adjusting fever intensity in real‐time. Additionally, microglia‐derived Caspase‐11 emerges as a novel regulator, offering another avenue for cycling control by toggling fever responses with molecular precision.^[^ ^193^ ^]^

### 4.2 Peripheral Pattern‐Induced

The non‐central mode of the febrile mechanism mainly involves the peripheral production of PGE2 by KCs in the liver. Transmitting signals to the brain via the hepatic vagus nerve, which regulates the thermoregulatory point and thus triggers the febrile response.^[^ ^32^, ^57^, ^59^ ^]^

Two innovative approaches drive controllable non‐central fever induction: 1) liver‐targeted engineered bacteria design and 2) spatiotemporal regulation of localized PGE2 release. The hepatic‐targeting engineered bacteria strategy involves modifying bacterial hepatotropism and complement‐activating capacity to mimic the natural hepatic accumulation process of LPS. The engineered bacteria directly stimulate KCs by secreting LPS analogs or complement‐activating factors, triggering complement‐dependent early‐phase fever signaling and thus integrating input control (via programmable bacterial design) with cycling control (via on‐demand activation) while also offering dual therapeutic potential by remodeling the tumor immune microenvironment.^[^ ^194^, ^195^ ^]^ The local PGE2 release strategy employs genetic engineering to reconstruct PGE2 biosynthesis pathways within KCs.^[^ ^184^ ^]^ For instance, enhanced PGE2 biosynthesis via CRISPR‐Cas9‐mediated metabolic reprogramming.^[^ ^196^ ^]^ The CRISPR‐Cas9 genome‐editing platform enables the targeted reprogramming of critical enzymatic nodes, such as COX‐2 and microsomal prostaglandin E synthase‐1, thereby amplifying the metabolic flux from arachidonic acid to PGE2. This biosynthetic cascade is governed by drug delivery systems, ensuring stringent spatiotemporal control over PGE2 production. The synthesized PGE2 induces fever via paracrine signaling, binding to EP3 receptors on hepatic vagal afferent nerve terminals.

The non‐central and central modes of fever induction are intricately intertwined. The engineered bacteria approach could be reengineered to synthesize systemic pyrogens (IL‐1β or TNF‐α), which engage the COX‐2‐PGE2 axis in brain endothelial cells to drive central fever responses. Likewise, the localized PGE2 release strategy, which employs CRISPR‐Cas9‐mediated reprogramming of peripheral enzymatic pathways, could be adapted to deliver PGE2 or its precursors directly to the hypothalamus, thereby effectively modulating thermoregulatory pathways.

In summary, advancements in bioengineering enable the controlled induction of EFT by precisely modulating molecular regulators. Control is achieved at three levels: i) input control, achieved through tailored modulation of molecular triggers such as nanoparticles and engineered bacteria; ii) process control, facilitated by real‐time monitoring and adjustment of fever dynamics; and iii) cycling control, allowing precise activation and deactivation of the fever response.

### 4.3 Clinical Safety Considerations in EFT

To fully address the controllability challenges of EFT, a dedicated examination of toxicity profiles and patient safety measures is essential. EFT can mimic physiological fever, driving systemic immune activation in oncology. However, prolonged fever‐range hyperthermia (typically 39–42 °C) can potentially trigger adverse events, including cytokine storms, cardiovascular strain, and CNS complications.^[^ ^197^, ^198^, ^199^, ^200^ ^]^ These risks arise from the overactivation of immune pathways, such as the excessive release of pro‐inflammatory cytokines (e.g., IL‐6, TNF‐α), which may lead to systemic inflammation and organ dysfunction.^[^ ^31^ ^]^ Clinical and preclinical investigations have documented adverse outcomes linked to elevated temperatures. Preclinical findings indicate that mice simultaneously exposed to 39.5 °C heat and subjected to a DNP‐induced temperature rise—which further elevated core body temperature by ≈1 °C (reaching 42.11 °C) via mitochondrial uncoupling—exhibited significant proteinuria and worsened renal injury.^[^ ^201^ ^]^ The degree of renal injury was strongly positively correlated with core body temperature (*R *= 0.715, *P *< 0.001). Moreover, Clinical studies demonstrate that fever is associated with significant adverse outcomes in critically ill patients. In sepsis patients admitted in sinus rhythm, fever‐linked sepsis‐induced coagulopathy correlates with new‐onset atrial fibrillation.^[^ ^202^ ^]^ A meta‐analysis (180 studies, 460825 acute brain injury patients) shows fever raises unfavorable neurological outcomes (OR 2.37), mortality (OR 1.31), deterioration (OR 1.10), delayed ischemia (OR 1.96), large infarcts (OR 2.94), hemorrhagic transformation (OR 1.63), and hemorrhage volume (OR 2.38).^[^ ^203^ ^]^ In pediatric populations, febrile seizures triggered by fever in 2–4% of children aged 6 months to 5 years lead to long‐term neurodevelopmental issues, including attention deficit hyperactivity disorder, epilepsy risk, and hippocampal sclerosis.^[^ ^204^ ^]^ To address these concerns, strategies for mitigation, along with patient selection and monitoring protocols, were outlined to improve EFT's clinical translatability.

Cytokine storms represent a primary toxicity risk in EFT, characterized by a hyperinflammatory response that can escalate to multi‐organ failure.^[^ ^198^ ^]^ To mitigate this, controllable EFT incorporates precision modulation techniques, such as nanoparticle‐mediated controlled release of fever inducers, enabling dose titration and temporal control to maintain cytokine levels within therapeutic windows (e.g., avoiding IL‐6 peaks above 100 pg mL^−1^).^[^ ^205^, ^206^ ^]^ Furthermore, integration with localized targeting enhances safety by confining initial heat application to tumors, thereby priming systemic responses without triggering overwhelming global cytokine release.^[^ ^207^ ^]^

Cardiovascular strain is another key concern, as hyperthermia increases heart rate, cardiac output, and vascular permeability, potentially exacerbating conditions like hypertension or ischemia.^[^ ^208^ ^]^ Employing biofeedback systems that integrate real‐time physiological monitoring with dynamic thermal regulation can relieve. For example, wearable devices equipped with photoplethysmography sensors continuously track heart rate variability and blood pressure. Meanwhile, feeding data into a closed‐loop system that can adjust EFT activation to maintain core temperature below a safe temperature (<41 °C).^[^ ^209^, ^210^ ^]^

CNS complications, such as seizures or neuroinflammation resulting from high fever, are mitigated by incorporating neuroprotective elements (e.g., Bruton's tyrosine kinase inhibitors) to regulate microglial activation.^[^ ^211^ ^]^ Controllable EFT platforms can use real‐time neuroimaging or EEG monitoring to detect early signs of CNS distress, allowing immediate intervention.^[^ ^212^, ^213^ ^]^

Patient selection is crucial for minimizing risks. Ideal candidates include those with intact immune function and no pre‐existing conditions such as cardiovascular disease, autoimmune disorders, or neurological impairments. Exclusion criteria should encompass patients with a history of cytokine release syndrome (e.g., from prior immunotherapies) or those over 65 years with comorbidities. Stratification based on biomarkers, such as baseline C‐reactive protein levels or genetic profiling for cytokine polymorphisms, can further refine selection. Additionally, patients with low baseline inflammation may tolerate higher fever intensities.^[^ ^205^ ^]^

Monitoring strategies form the backbone of safe EFT implementation. Continuous vital sign tracking via wearable sensors (e.g., for core temperature, heart rate variability, and blood pressure) enables dynamic adjustments.^[^ ^210^, ^214^ ^]^ Laboratory monitoring of cytokine panels (e.g., IL‐6, TNF‐α) and cardiac biomarkers (e.g., troponin) is performed every 30 min during induction, detecting early aberrations. Advanced protocols include artificial intelligence (AI)‐driven predictive algorithms to forecast storm onset based on real‐time data, allowing preemptive dose reduction.^[^ ^198^, ^215^ ^]^ For instance, a study analyzing high‐frequency temperature monitoring data from 68 hospitalized patients undergoing hematopoietic stem cell transplantation or CAR‐T therapy developed an advanced computer algorithm to fit fever rhythms, enabling early prediction of febrile adverse events.^[^ ^215^ ^]^

By incorporating these measures, EFT not only addresses its core controllability challenge but also paves the way for safer clinical translation, potentially reducing adverse event rates similar to those observed in hyperthermia trials. Future studies should validate these strategies in Phase I trials to confirm their efficacy in diverse patient populations.

## 5 Conclusions and Prospects

Fever functions as a potent immunomodulator, enhancing immune cell metabolism, promoting immune cell trafficking, and regulating the differentiation of innate and adaptive immune cells, positioning it as a promising adjuvant strategy for tumor therapy.^[^ ^21^, ^74^, ^216^, ^217^ ^]^ As highlighted earlier, EFT has demonstrated significant therapeutic potential in clinical settings, achieving favorable outcomes in tumor treatment and improving patient prognosis.^[^ ^24^, ^92^, ^218^ ^]^ Its capacity to induce systemic immune activation offers distinct advantages, particularly in addressing metastatic tumors and challenging malignancies.^[^ ^8^, ^9^, ^11^, ^12^, ^71^ ^]^ Key benefits of EFT include: i) sustaining fever‐range temperatures within the tumor microenvironment to enhance immune cell recruitment and activation while mitigating heat‐induced immunosuppressive feedback, thereby fostering durable anti‐tumor responses;^[^ ^12^, ^21^, ^219^ ^]^ and ii) mobilizing systemic immunity to target metastatic and micrometastatic lesions, potentially enabling comprehensive tumor eradication.^[^ ^167^, ^220^ ^]^ Given these systemic immune effects, EFT is not only a standalone therapy but also a synergistic complement to chemotherapy, immunotherapy, and targeted therapies, amplifying overall therapeutic efficacy.

Despite its promise, the current clinical adoption of EFT has been limited due to advancements in immunotherapy and persistent safety concerns associated with traditional fever induction methods. Among them, bacterial extract‐induced fever often triggers excessive immune responses, manifesting as severe inflammation, erythema, and edema.^[^ ^221^ ^]^ Drug‐induced fevers exhibit significant inter‐individual variability, complicating standardization and compromising reliability.^[^ ^15^ ^]^ Furthermore, unpredictable temperature fluctuations in these approaches pose safety risks and diminish therapeutic precision. To overcome the challenges hindering the clinical translation of EFT and to harness its therapeutic potential, future research should focus on several strategic directions:

**i) Deepening Mechanistic Insights**: Advancing the understanding of human fever pathways to identify precise regulatory targets is critical for achieving controllable fever induction with robust input control. This involves elucidating molecular mechanisms, such as the thermomiR‐RBM3 interplay and COX‐2‐PGE2 signaling, to enable targeted interventions that initiate fever responses with precision.

**ii) Innovating Bioengineering Solutions**: Leveraging smart nanomaterials and bioengineering technologies, including liver‐targeted engineered bacteria and CRISPR‐Cas9‐mediated PGE2 modulation, will facilitate the development of EFT strategies that ensure accurate temperature and immune response modulation. These approaches enable process control by dynamically adjusting fever intensity and duration through feedback‐driven systems, ensuring alignment with therapeutic objectives while promoting quantitative reprogramming of immune cells to enhance systemic anti‐tumor immunity. Additionally, engineering design EFT to preferentially recruit tumor‐specific adaptive immune cells (e.g., T or B cells with antigen‐specific receptors) could enhance the precision of quantitative immune reprogramming. For example, multimodal programmable biological platforms that integrate fever‐inducing agents with CAR technology, or nano‐antibodies (e.g., anti‐CD3), may promote selective T‐cell infiltration via chemokine gradients.^[^ ^222^, ^223^, ^224^ ^]^ Similarly, for B cells, EFT might incorporate antigen‐specific adjuvants using a nano‐delivery system to promote germinal center formation and affinity maturation, thereby promoting the activation of B cells with tumor antigen‐specific receptors and their effector functions in the tumor microenvironment.^[^ ^225^, ^226^, ^227^ ^]^

**iii) Exploring Synergistic Integrations**: Investigating the synergistic potential of EFT with immunotherapies, such as immune checkpoint inhibitors and cellular therapies, will amplify anti‐tumor efficacy. A feasible strategy is to refer to the existing whole‐body hyperthermia combination.^[^ ^162^, ^163^, ^171^ ^]^ EFT initially induces fever to activate the immune system, maintaining a controlled temperature of 39–41 °C with continuous monitoring of stable body temperature and vital signs. Upon confirming the absence of adverse reactions, combined immunotherapy is administered, incorporating checkpoint inhibitors (anti‐PD‐1/PD‐L1) or CAR‐T cell therapy. The treatment sequence, whether EFT precedes immunotherapy or vice versa, requires further exploration in future preclinical or clinical studies. EFT is expected to complement existing therapies, amplifying their effectiveness while minimizing the risks associated with uncontrolled fever induction.

**iv) AI Empowerment in Controllable EFT**: The integration of AI holds transformative potential for advancing controllable EFT by enhancing precision, personalization, and scalability. AI‐driven predictive models can leverage multimodal data, including real‐time temperature profiles, immune cell dynamics, and patient‐specific genomic information to optimize fever induction parameters, ensuring tailored temperature ranges and durations that maximize individual therapeutic efficacy.^[^ ^198^, ^205^ ^]^ Furthermore, AI‐enabled closed‐loop systems, incorporating biosensors and real‐time feedback mechanisms, can dynamically modulate fever intensity, mitigating adverse reactions and improving safety profiles.^[^ ^209^, ^210^, ^214^ ^]^ AI‐powered computational simulations can accelerate preclinical validation by modeling the synergistic effects of EFT with immunotherapies, predicting long‐term therapeutic outcomes, and informing the design of robust multicenter clinical trials. In clinical settings, establishing standardized clinical protocols through large‐scale, multicenter prospective trials is essential to define optimal fever temperature ranges (38.5–40 °C) and durations (4–6 h). These protocols will provide robust evidence to support EFT's widespread clinical adoption, ensuring enhanced safety through controlled fever dynamics and paving the way for its integration into precision oncology.

This review presents a novel perspective on controllable EFT, aiming to catalyze its advancement for future disease treatment by integrating febrile immunomodulation with thermal immunotherapy. The realization of EFT's clinical potential hinges on precise fever induction with input and process control, quantitative reprogramming of immune responses, and optimized fever dynamics. The successful translation of controllable EFT into clinical practice will require interdisciplinary collaboration among researchers, bioengineers, and clinicians. Advances in nanotechnology, molecular engineering, and precision medicine will be pivotal in optimizing EFT's safety profile, refining its engineering techniques, and validating its long‐term efficacy. By overcoming the technical and biological barriers, multidisciplinary efforts can transform EFT into a cornerstone of cancer therapy, harnessing its immunomodulatory potential to enhance patient outcomes in precision oncology.

## Conflict of Interest

The authors declare no conflict of interest.
